# Supplementary material for: G2Retro as a two-step graph generative models for retrosynthesis prediction
Source: Commun Chem. 2023 May 30;6:102. doi: 10.1038/s42004-023-00897-3 (PMC10229662; doi:10.1038/s42004-023-00897-3)
Supplement: Supplementary file 2 — Supplementary Information [file 42004_2023_897_MOESM2_ESM.pdf]

G[2]Retro as a Two-Step Graph Generative Model for Retrosynthesis Prediction

Ziqi Chen[1], Oluwatosin R. Ayinde[2], James R. Fuchs[2], Huan Sun[1,3], Xia Ning[1,3,4] ✉

Supplementary Note 1: G²Retro with fragments: G²Retro-B

Inspired by the recent success of using fragments in other tasks,[1] we extended G²Retro by incorporating the fragments generated from the breaking retrosynthetically interesting chemical substructures (BRICS) fragmentation algorithm[2], and denote the new method as G²Retro-B. BRICS breaks synthetically accessible bonds in a product M_p, following a set of fragmentation rules. Thus, the fragments generated from BRICS encode prior knowledge related to synthesis. G²Retro-B integrates such knowledge by learning from the molecular graph constructed from the fragments. Specifically, for each M_p, G²Retro-B constructs a BRICS graph $\mathcal{G}_p^B = (\mathcal{V}, \mathcal{E})$, where each node $n_u \in \mathcal{V}$ represents a BRICS fragment with all the atoms and bonds belonging to it, and each edge $e_{uv} \in \mathcal{E}$ corresponds to a bond b_{ij} that connects two BRICS fragments n_u and n_v. That is, the two atoms connected by b_{ij} belong to n_u and n_v, respectively (i.e., $a_i \in n_u$ and $a_j \in n_v$; In our dataset, two BRICS fragments are connected through only one bond). Thus, $\mathcal{E}$ includes synthetically accessible bonds, which tend to be the reaction centers, and thus, $\mathcal{G}_p^B$ incorporates fragment-level structures of M_p. For simplicity, when no ambiguity arises, we omit the super/sub-scripts and use $\mathcal{G}^B$ to represent $\mathcal{G}_p^B$.

G²Retro-B generates BRICS fragment embeddings by passing the messages along the connections over BRICS fragments in the BRICS graphs, in a similar way as for atom embeddings over molecular graphs. Specifically, each edge e_{uv} in $\mathcal{G}^B$ is associated with two message vectors $\mathbf{e}_{uv}$ and $\mathbf{e}_{vu}$. The message $\mathbf{e}_{uv}^{(t)}$ at t-th iteration is updated as follows,

$$\mathbf{e}_{uv}^{(t)} = W_1^e \text{ReLU}(W_2^e \mathbf{s}_u + W_3^e \mathbf{s}_{uv} + W_4^e \sum_{n_w \in \mathcal{N}(n_u) \setminus \{n_v\}} \mathbf{e}_{wu}^{(t-1)}), \tag{S1}$$

where $\mathbf{s}_u = \sum_{a_i \in n_u} \mathbf{a}_i$ aggregates the embeddings of all the atoms within the fragment n_u; $\mathbf{s}_{uv} = \mathbf{a}_i$ is the embedding of atom a_i in n_u that is included in the edge e_{uv}; W_i^e's (i=1,2,3,4) are the learnable parameter matrices; $\mathbf{e}_{uv}^{(0)}$ is initialized with the zero vector. The message $\mathbf{e}_{uv}^{(t)}$ encodes the information passing through the edge e_{uv} to n_v, and thus is used to further derive the embedding of n_v as follows,

$$\mathbf{n}_v = U_1^e \text{ReLU}(U_2^e \mathbf{s}_v + U_3^e \sum_{n_w \in \mathcal{N}(n_v)} \mathbf{e}_{wv}^{(1 \cdots t_e)}), \tag{S2}$$

where $\mathbf{e}_{wv}^{(1 \cdots t_e)}$ denotes the concatenation of $\{\mathbf{e}_{wv}^{(t)} | t \in [1 : t_e]\}$; U_i^e's (i=1,2,3) are the learnable parameter matrices. With $\mathcal{G}^B$, G²Retro-B enriches the representation of atom a_i with the embedding $\mathbf{n}_v$ of the fragment that a_i belongs to. Note that in BRICS algorithm, each atom only belongs to one fragment. The enriched atom representation is calculated as follows,

$$\mathbf{a}'_i = V(\mathbf{a}_i \oplus \mathbf{n}_v), \tag{S3}$$

where V is a learnable hyperparameter matrix; $\oplus$ represents the concatenation operation.

The reaction center identification in G²Retro-B is done in the same way as in G²Retro (Section "Reaction Center Identification" in the main manuscript), with all the enriched atom representations calculated as above, and bond embeddings (e.g., Equation 7 in the main manuscript) calculated using the enriched atom representations. Note that synthon completion in G²Retro-B does not use the BRICS graph and thus is identical to G²Retro.

Supplementary Note 2: G²Retro ensemble: G²Retro-ens

To explore a large reaction space, we developed an ensemble approach for G²Retro, denoted as G²Retro-ens. G²Retro-ens ensembles 20 G²Retro models that are combined from the top-4 reaction center identification modules and the top-5 synthon completion modules, each selected based on the corresponding validation data (hyper-parameter space follows that in Supplementary Table 3 except for atom embedding dimensions in {32, 64}). For each target product, all the top-10 predicted reactions from the 20 G²Retro models are combined based on their average ranking (different G²Retro may predict the same reaction), and the final top-10 predicted reactions are considered as the results of G²Retro-ens.

Supplementary Table 1 presents the performance comparison between G²Retro-ens and R–SMILES on different reaction types. Among the 10 reaction types in the benchmark data, G²Retro-ens outperforms R–SMILES at top-1 accuracy on 5 reaction types, and archives the same performance on 2 reaction types. On average, G²Retro-ens outperforms R–SMILES on the most popular reaction types on higher-ranked predictions (i.e., corresponding to smaller k in top-k accuracy). For example, G²Retro-ens substantially outperforms R–SMILES on deprotection reactions (54.2% vs 52.7% on top-1 accuracy).

[1]Computer Science and Engineering, The Ohio State University, Columbus, OH 43210, USA. [2]Medicinal Chemistry and Pharmacognosy, College of Pharmacy, The Ohio State University, Columbus, OH 43210, USA. [3]Translational Data Analytics Institute, The Ohio State University, Columbus, OH 43210, USA. [4]Biomedical Informatics, The Ohio State University, Columbus, OH 43210, USA. ✉ning.104@osu.edu

Supplementary Table 1. Performance comparison between G²Retro-ens and R–SMILES on different reaction types

Type Name	Percentage (%)	G²Retro-ens				R–SMILES			
		1	3	5	10	1	3	5	10
heteroatom alkylation and arylation	30.3	56.5	80.7	88.5	94.4	56.5	81.3	88.1	93.5
acylation and related processes	23.8	69.7	91.1	95.4	98.2	68.7	89.8	93.9	96.4
deprotections	16.5	54.2	80.1	87.1	92.4	52.7	76.6	81.4	86.7
C-C bond formation	11.3	41.4	64.2	71.4	80.6	39.7	63.5	74.3	81.7
reductions	9.2	61.0	78.4	84.6	90.7	59.3	80.1	87.7	92.2
functional group interconversion	3.7	35.3	57.1	67.4	73.4	42.4	57.6	66.3	79.3
heterocycle formation	1.8	0.0	0.0	0.0	0.0	48.4	70.3	78.0	83.5
oxidations	1.6	68.3	86.6	90.2	93.9	54.9	82.9	92.7	97.6
protections	1.4	51.5	77.9	85.3	89.7	58.8	82.4	88.2	91.2
functional group addition	0.5	78.3	87.0	87.0	95.7	78.3	87.0	91.3	95.7

Columns with 1, 3, 5 and 10 present top-1, top-3, top-5 and top-10 accuracies, respectively. Column "Percentage(%)" represents the percentage of reactions in the test set belonging to the specific reaction type.

Supplementary Note 3: Additional case study

For Mavacamten as in Supplementary Figure **1a**, which was approved by FDA in 2022 to treat hypertrophic cardiomyopathy,[3] the patent literature[4] reports the utilization of a nucleophilic aromatic substitution for the formation of the C8-N9 bond (ground truth in Supplementary Figure **1b**).G²Retro correctly predicts this coupling as the top-1 reaction (Supplementary Figure **1c**), and also identifies its additional permutations by replacing the aryl chloride with the aryl bromide and the aryl fluoride, respectively (Supplementary Figure **1d** and **1g**). Aryl fluorides in Supplementary Figure **1g** are not as typical as aryl chlorides and bromides, and G²Retro ranks the substitution reaction involving the arly fluoride low. In addition to the amine coupling strategy with aryl halides, G²Retro also identifies the reaction of the amine with trifluoro methyl sulfate to make the same bond (Supplementary Figure **1h**), which would be expected to work as with aryl halides in Supplementary Figure **1c** and **1d**. However, the alcohol in Supplementary Figure **1i** is not a good enough leaving group to make the bond (i.e., C8-N9). Interestingly, G²Retro also identifies other amine linkages (e.g., in Supplementary Figure **1e** between C2 and N4; in Supplementary Figure **1j** between N9 and C10) as potential reaction centers. However, the proposed synthesis in Supplementary Figure **1e** and **1f** would most likely lead to the formation of undesired products as these reactant pairs would likely result in the alkylation of both N4 and N9. Therefore, the use of the aryl halides in Supplementary Figure **1c** and **1d** would be the more efficient way of obtaining the desired product.

Oteseconazole as in Supplementary Figure **2a** is a drug approved for recurrent vulvovaginal candidiasis[5]. In the patent literature[6], this drug is constructed by the C-C bond forming reaction between C6 and C7 and is assembled with Suzuki coupling[7] between an aryl bromide group and a boronic ester (Supplementary Figure **2b**). G²Retro correctly predicts this coupling as the top-1 with the boronic acid (Supplementary Figure **2c**), the top-3 which is the same as the patented reaction (Supplementary Figure **2e**), and the top-9 reaction with a relatively uncommon boronic ester (Supplementary Figure **2k**). Boronic acids in Supplementary Figure **2c** would typically be considered by synthetic chemists as interchangeable with boronic esters, and thus should be considered a feasible reaction; while Boronic ester in Supplementary Figure **2k** should react in the same way with the patented reaction, and thus could deliver the desired compound. Interestingly, G²Retro also predicts the Ullmann-type coupling[8] with different aryl halides to construct the C6-C7 bond in Supplementary Figure **2d**, **2f** and **2g**, all of which would be expected as feasible reactions. Although the reaction center is correctly identified in Supplementary Figure **2i**, the proposed coupling of two boronic acids would not be effective. G²Retro also identifies another C-N coupling of various aryl halides with imidazoles (C15-N16 - Supplementary Figure **2h** and **2j**), which hypothetically would also work as expected.

Supplementary Note 4: Clustering algorithms for diversity analysis

Supplementary Algorithm 1 describes the algorithm to cluster products for diversity analysis. Given K products $\{M_p^k\}_{k=1,\cdots,K}$ and their top-10 predicted reactions $\{\{R_i^k\}_{i=1,\cdots,10}\}_{k=1,\cdots,K}$, we clustered products according to their reaction similarity distributions.

Supplementary Note 5: Algorithms of G²Retro

Supplementary Algorithm 2 describes the reactant generation process of G²Retro. Given a product, the maximum number of synthons K, the beam size N, and the maximum number of steps allowed maxSteps, G²Retro generate a ranked list of N reactants that can be used to synthesize the product. Supplementary Algorithm 3 describes how G²Retro converts the product graph into top-K synthon graphs. Given a product graph $\mathcal{G}_p$, its corresponding BRICS graph $\mathcal{G}^B$ and K, G²Retro predicts the top-K synthon graphs and calculates their log-likelihood scores $\{s_k\}$, using the learned molecule representations from the encoder described in Supplementary Algorithm 4. Specifically, G²Retro first selects the top-K most possible reaction centers and calculates their log-likelihood scores $\{s_k, C_k\}_{k=1}^K$. Then given the product graph, the top-K reaction centers and their scores and the product molecule representation $\mathbf{h}_p$, G²Retro transforms the product graph into top-K synthon graphs as in Supplementary Algorithm 5. Supplementary Algorithm 6 describes how G²Retro completes top-K synthon graphs into top-N reactant graphs. Given the product graph $\mathcal{G}_p$, the top-K synthon graphs and their scores $\{s_k, \mathcal{G}_{s,k}\}_{k=1}^K$, the beam size N, and the maximum number of completion steps maxSteps, G²Retro uses a beam

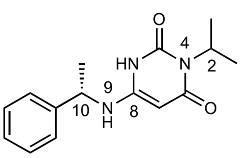

1a, Mavacamten

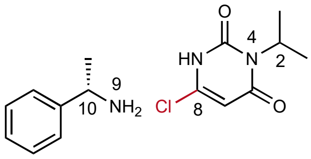

1b, ground-truth reactants

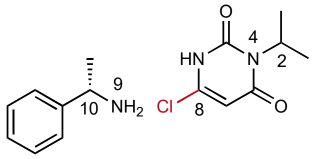

1c, top-1 predicted reactants

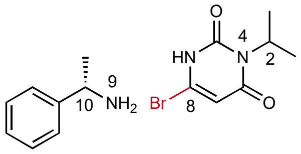

1d, top-2 predicted reactants

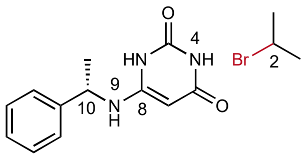

1e, top-3 predicted reactants

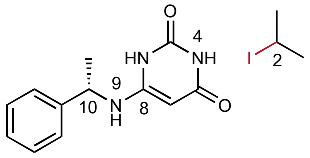

1f, top-4 predicted reactants

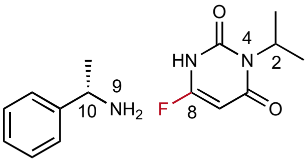

1g, top-5 predicted reactants

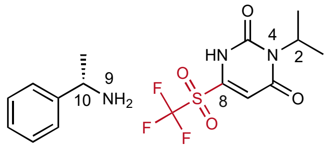

1h, top-6 predicted reactants

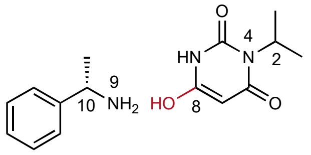

1i, top-7 predicted reactants

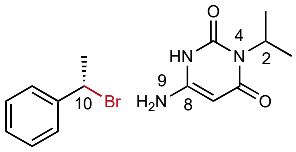

1j, top-8 predicted reactants

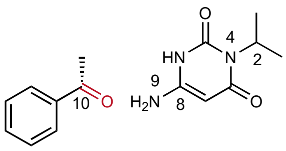

1k, top-9 predicted reactants

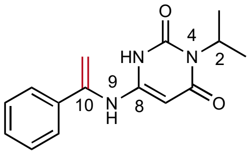

1l, top-10 predicted reactants

Supplementary Figure 1. Predicted reactions by $\mathsf{G^2Retro}$ **for "mavacamten"**. Numbers next to each atom are the indices of the atoms. Atoms with same indices in different subfigures are corresponding to each other. Atoms and bonds colored in red are leaving groups for synthon completion. **1a**, product/target molecule; **1b**, the ground-truth reactants in USPTO-50K; **1c-1l**, top predicted reactants.

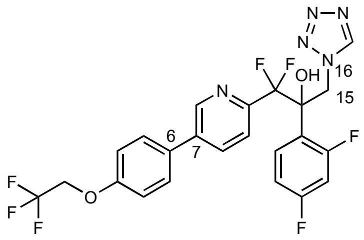

2a, Oteseconazole

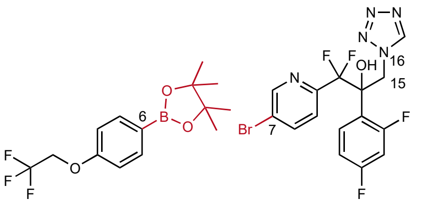

2b, ground-truth reactants

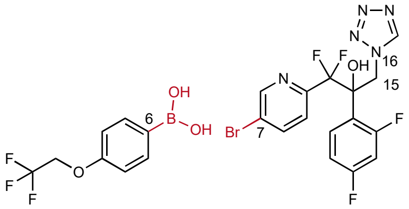

2c, top-1 predicted reactants

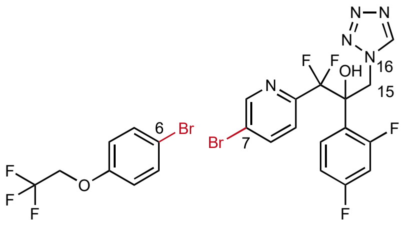

2d, top-2 predicted reactants

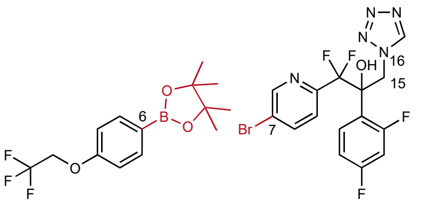

2e, top-3 predicted reactants

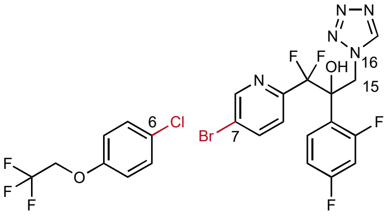

2f, top-4 predicted reactants

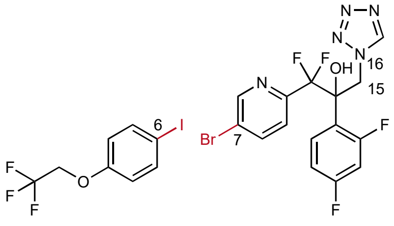

2g, top-5 predicted reactants

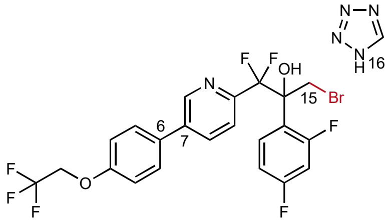

2h, top-6 predicted reactants

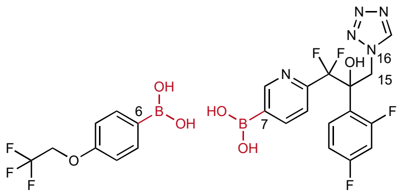

2i, top-7 predicted reactants

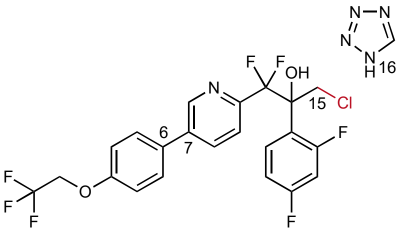

2j, top-8 predicted reactants

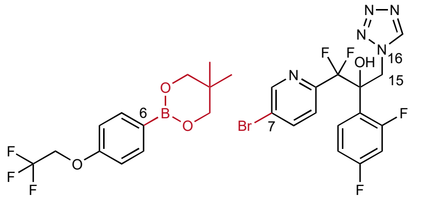

2k, top-9 predicted reactants

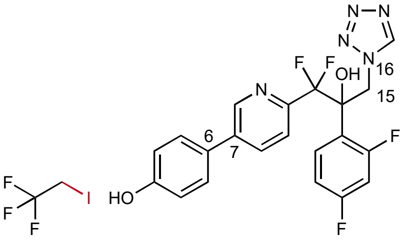

2l, top-10 predicted reactants

Supplementary Figure 2. Predicted reactions by $\mathsf{G^2Retro}$ **for Oteseconazole**. Numbers next to each atom are the indices of the atoms. Atoms with same indices in different subfigures are corresponding to each other. Atoms and bonds colored in red are leaving groups for synthon completion. **2a**, product/target molecule; **2b**, the ground-truth reactants in USPTO-50K; **2c-2l**, top predicted reactants.

search strategy to complete the synthon graphs into the reactant graphs in a sequential way. Supplementary Algorithm 7 describes the beam search strategy. Given the queue of intermediate molecules Q, the queue of completed reactants R, the representations of top-K synthons $\{\mathbf{h}_{s,k}\}_{k=1}^{K}$, the product representation $\mathbf{h}_p$, and the beam size N, $\mathsf{G^2Retro}$ extends

Supplementary Algorithm 1 Clustering products according to reaction similarity distributions

Require: $\{M_p^k, \{R_i^k\}_{i=1,\cdots,10}\}_{k=1,\cdots,K}$, NClusters

for each M_p^k and $\{R_i^k\}_{i=1,\cdots,10}$ **do**
  ▷ calculate pair-wise similarities among top-10 predictions (Equation 1)
  for each pair of reactions (R_i^k, R_j^k) with $i \neq j$ **do**
    $s_{ij}^k = \text{sim}(R_i^k, R_j^k)$
  end for
  ▷ generate the reaction similarity distribution of product M_p^k
  $\mathbf{h}^k = \text{histogram}(\{s_{ij}^k\}_{\forall i,j})$
end for
▷ cluster products using K-Means according to their reaction similarity distributions
$\{C_i\}_{i=1,\cdots,\text{NClusters}} = \text{K-Means}(\{\mathbf{h}^k\}_{k=1,\cdots,K}, \text{NClusters})$
return $\{C_i\}_{i=1,\cdots,\text{NClusters}}$

each intermediate molecule in the queue by attaching different substructures at the attachment point, and saves the completed molecules into R and the incomplete ones for the next completion step. Supplementary Algorithm 8 describes how to attach new substructures to an intermediate molecule using the Atom Attachment Continuity Prediction (AACP) and the Atom Attachment Type Prediction (AATP).

Supplementary Algorithm 2 $\mathsf{G^2Retro}$

Require: $M_p = (\mathcal{G}_p, \mathcal{G}_p^B)$, K, N, maxSteps
▷ predict top-K synthons with Supplementary Algorithm 3
$\{s_k, \mathcal{G}_{s,k}\}_{k=1}^K = \mathsf{G^2Retro\text{-}RCI}(\mathcal{G}_p, \mathcal{G}_p^B, K)$
▷ predict top-N reactants with Supplementary Algorithm 6
$\{\mathcal{G}_{r,i}\}_{i=1}^N = \mathsf{G^2Retro\text{-}SC}(\mathcal{G}_p, \{s_k, \mathcal{G}_{s,k}\}_{k=1}^K, N, \text{maxSteps})$
return $\{\mathcal{G}_{r,i}\}_{i=1}^N$

Supplementary Algorithm 3 $\mathsf{G^2Retro\text{-}RCI}$ for Reaction Center Identification

Require: $\mathcal{G}_p$, $\mathcal{G}^B$, K
▷ learn molecule representations with Supplementary Algorithm 4
$\{\mathbf{a}_i\}, \{\mathbf{b}_{ij}\}, \mathbf{h}_p = \mathsf{G^2Retro\text{-}encoder}(\mathcal{G}_p, \mathcal{G}^B)$
▷ select top-K BF-centers (Equation 8)
$\{s^b(b_{ij})\}^K = \text{top}(K, \text{findCenter}(\mathsf{BF\text{-}center}, \{\mathbf{b}_{ij}\}, \mathbf{h}_p))$
▷ select top-K BC-centers (Equation 11)
$\{s_k^c(b_{ij})\}^K = \text{top}(K, \text{findCenter}(\mathsf{BC\text{-}center}, \{\mathbf{b}_{ij}\}, \mathbf{h}_p))$
▷ select top-K A-centers (Equation 12)
$\{s^a(a_i)\}^K = \text{top}(K, \text{findCenter}(\mathsf{A\text{-}center}, \{\mathbf{a}_i\}, \mathbf{h}_p))$
▷ select top-K centers $\{C_k\}$ and calculate their log-likelihoods $\{s_k\}$
$\{s_k, C_k\}_{k=1}^K = \text{top}(K, \{s^b(b_{ij})\}^K, \{s_k^c(b_{ij})\}^K, \{s^a(a_i)\}^K)$
▷ convert a product into K sets of synthons and update their log-likelihoods with Supplementary Algorithm 4
$\{s_k, \mathcal{G}_{s,k}\}_{k=1}^K = \mathsf{G^2Retro}\text{-}p2s\text{-}\mathsf{T}(\mathcal{G}_p, \{s_k, C_k\}_{k=1}^K, \mathbf{h}_p)$
return $\{s_k, \mathcal{G}_{s,k}\}_{k=1}^K$

Supplementary Algorithm 4 G^{2}Retro-encoder

Require: $\mathcal{G}$, $\mathcal{G}^B$

▷ calculate atom embeddings
$\{\mathbf{a}_i\} = \mathsf{GMPN}(\mathcal{G})$
▷ calculate the graph embedding (Equation 6)
$\mathbf{h} = \sum_{a_i \in \mathcal{G}} \mathbf{a}_i$
if use BRICS **then**
  $\{\mathbf{n}_u\} = \mathsf{FMPN}(\mathcal{G}^B, \{\mathbf{a}_i\})$
  ▷ update the embedding of each atom with its BRICS fragment embedding (Equation S3)
  $\{\mathbf{a}_i\} = \{V(\mathbf{a}_i \oplus \mathbf{n}_u)\}$
end if
▷ calculate bond embeddings (Equation 7)
$\{\mathbf{b}_{ij}\} = \text{bondEmb}(\{\mathbf{a}_i\})$
return $\{\mathbf{a}_i\}$, $\{\mathbf{b}_{ij}\}$, $\mathbf{h}$

Supplementary Algorithm 5 G^{2}Retro-$p2s$-T for transformation from product to synthons

Require: $\mathcal{G}_p$, $\{s_k, C_k\}_{k=1}^K$, $\mathbf{h}_p$

for each s_k, C_k **do**
  if C_k is BF-center **then**
    ▷ predict bonds with induced type changes and calculate the log-likelihood s_{BF} for the predictions of $\mathcal{C}_{\mathsf{BF}}(C_k)$
    $\mathcal{C}_{\mathsf{BF}}{}'(C_k), s_{\mathsf{BF}} = \mathsf{BTCP}(C_k, \mathcal{C}_{\mathsf{BF}}(C_k), \mathbf{h}_p)$
    ▷ add the predicted bonds with type changes into the center
    $C_k = C_k \cup \mathcal{C}_{\mathsf{BF}}{}'(C_k)$
    ▷ update the log-likelihood score
    $s_k = s_k + s_{\mathsf{BF}}$
  end if
  ▷ predict atoms with charge changes for all the atoms within the center $\mathcal{C}_{\mathsf{A}}(C_k)$, and calculate the log-likelihood score s_{A} for the predictions of $\mathcal{C}_{\mathsf{A}}(C_k)$
  $\mathcal{C}_{\mathsf{A}}{}'(C_k), s_{\mathsf{A}} = \mathsf{ACP}(C_k, \mathcal{C}_{\mathsf{A}}(C_k), \mathbf{h}_p)$
  ▷ update the log-likelihood score
  $s_k = s_k + s_{\mathsf{A}}$
  ▷ transform the product graph into the synthon graph with reaction center C_k and atom charge change $\mathcal{C}_{\mathsf{A}}{}'(C_k)$
  $\mathcal{G}_{s,k} = \text{transform}(\mathcal{G}_p, C_k, \mathcal{C}_{\mathsf{A}}{}'(C_k))$
end for
return $\{s_k, \mathcal{G}_{s,k}\}_{k=1}^K$

Supplementary Algorithm 6 $\mathsf{G^2Retro\text{-}SC}$ for synthon completion

Require: $\mathcal{G}_p$, $\{s_k, \mathcal{G}_{s,k}\}_{k=1}^K$, N, maxSteps

$t = 0$
▷ learn molecule representations with Supplementary Algorithm 4
-, -, $\mathbf{h}_p = \mathsf{G^2Retro}$-encoder$(\mathcal{G}_p)$
-, -, $\{\mathbf{h}_{s,k}\}_{k=1}^K = \mathsf{G^2Retro}$-encoder$(\{\mathcal{G}_{s,k}\}_{k=1}^K)$
▷ initialize a priority queue with synthons $\{\mathcal{G}_{s,k}\}_{k=1}^K$ as elements and $\{s_k\}_{k=1}^K$ as their priorities
$Q^{(0)} = \text{priorityQueue}(\{s_k, \mathcal{G}_{s,k}\}_{k=1}^K)$
▷ initialize an empty priority queue to store complete reactants
$R = \text{priorityQueue}()$
while $!Q^{(t)}$.isEmpty() and $t \leq$ maxSteps **do**
  ▷ stop the completion when it is impossible to get reactants better than the top-N reactants in R
  if R.size() $\geq N$ and R.nthLargestPriority$(N) \geq Q^{(t)}$.maxPriority() **then**
    break
  end if
  ▷ complete synthons through beam search with Supplementary Algorithm 7
  $Q^{(t+1)}, R = \mathsf{G^2Retro}$-beam-search$(Q^{(t)}, R, \{\mathbf{h}_{s,k}\}_{k=1}^K, \mathbf{h}_p, N)$
  $t = t + 1$
end while
▷ output top-N reactants
$\{\mathcal{G}_{r,i}\}_{i=1}^N = R$.nLargest$(N)$
return $\{\mathcal{G}_{r,i}\}_{i=1}^N$

Supplementary Algorithm 7 $\mathsf{G^2Retro}$-beam-search

Require: Q, R, $\{\mathbf{h}_{s,k}\}_{k=1}^K$, $\mathbf{h}_p$, N

$I = Q$.size()
$Q' = \text{priorityQueue}()$
while !Q.isEmpty() **do**
  $s_i, \mathcal{G}_i^* = Q$.pop()
  ▷ get the index of the synthon corresponding to $\mathcal{G}_i^*$
  $k = \mathcal{G}_i^*$.getSynthonIdx()
  ▷ predict the atom attachment for $\mathcal{G}_i^*$ with Supplementary Algorithm 8
  $\{s_{i,j}, \mathcal{G}'_{i,j}\}_{j=1}^{N+1} = \mathsf{G^2Retro}$-AAP$(\mathcal{G}_i^*, s_i, \mathbf{h}_{s,k}, \mathbf{h}_p, N)$
end while
▷ select top-N intermediate graph candidates
$\{s_i, \mathcal{G}'_i\}_{i=1}^N = \text{top}(N, \{\{s_{i,j}, \mathcal{G}'_{i,j}\}_{j=1}^{N+1}\}_{i=1}^I)$
for each s_i, $\mathcal{G}'_i$ **do**
  if $\mathcal{G}'_i$.isComplete() **then**
    R.push$(s_i, \mathcal{G}'_i)$
  else
    Q'.push$(s_i, \mathcal{G}'_i)$
  end if
end for
return Q', R

Supplementary Algorithm 8 $\mathsf{G^2Retro\text{-}AAP}$ for atom attachment prediction

Require: $\mathcal{G}^*$, s, $\mathbf{h}_s$, $\mathbf{h}_p$, N

$\triangleright$ get the atom that new substructures will be attached to
1: $a = \mathcal{G}^*.\text{nextAttachmentPoint}()$
$\triangleright$ predict whether further attachment should be added to a (Equation 18)
2: $s^o, s^{\neg o} = \mathsf{AACP}(a, \mathbf{h}_s, \mathbf{h}_p)$
$\triangleright$ extend $\mathcal{G}^*$ to the candidate $\mathcal{G}'_1$ that is predicted to stop at a
3: $\mathcal{G}'_1 = \text{stop}(\mathcal{G}^*, a)$
$\triangleright$ update the log-likelihood value of $\mathcal{G}'_1$
4: $s'_1 = s + s^{\neg o}$
$\triangleright$ predict the top-N new substructure attachments (Equation 20)
5: $\{z_i, s^z_i\}_{i=1}^N = \text{top}(N, \mathsf{AATP}(a, \mathbf{h}_s, \mathbf{h}_p))$
$\triangleright$ extend $\mathcal{G}^*$ to the candidates $\{\mathcal{G}'_i\}_{i=2}^{N+1}$ with the top-N substructures
6: $\{\mathcal{G}'_i\}_{i=2}^{N+1} = \text{attach}(\mathcal{G}^*, \{z_i\}_{i=1}^N)$
$\triangleright$ update the log-likelihood values of $\{\mathcal{G}'_i\}_{i=2}^{N+1}$
7: $\{s'_i\}_{i=2}^{N+1} = \{s + s^o + s^z_i\}_{i=1}^N$
8: **return** $\{s'_i, \mathcal{G}'_i\}_{i=1}^{N+1}$

Supplementary Note 6: Notations

Supplementary Table 2. Notations

Notation	Meaning
$M_r/M_s/M_p$	reactant/synthon/product molecule
$\mathcal{G} = (\mathcal{A}, \mathcal{B})$	molecular graph with atoms $\mathcal{A}$ and bonds $\mathcal{B}$
a	an atom in $\mathcal{G}$
b_{ij}	a bond in $\mathcal{G}$ connecting a_i and a_j
$\mathbf{x}$	a feature vector for an atom or a bond
$\mathcal{C}_{\mathsf{BF}}$	a set of bonds neighboring the bond formation center
$\mathcal{C}_{\mathsf{A}}$	a set of atoms within the reaction center
z	a substructure used to complete synthons into reactants
$\mathcal{Z}$	a vocabulary with all the substructures in the dataset

Supplementary Note 7: Substructures used to complete synthons

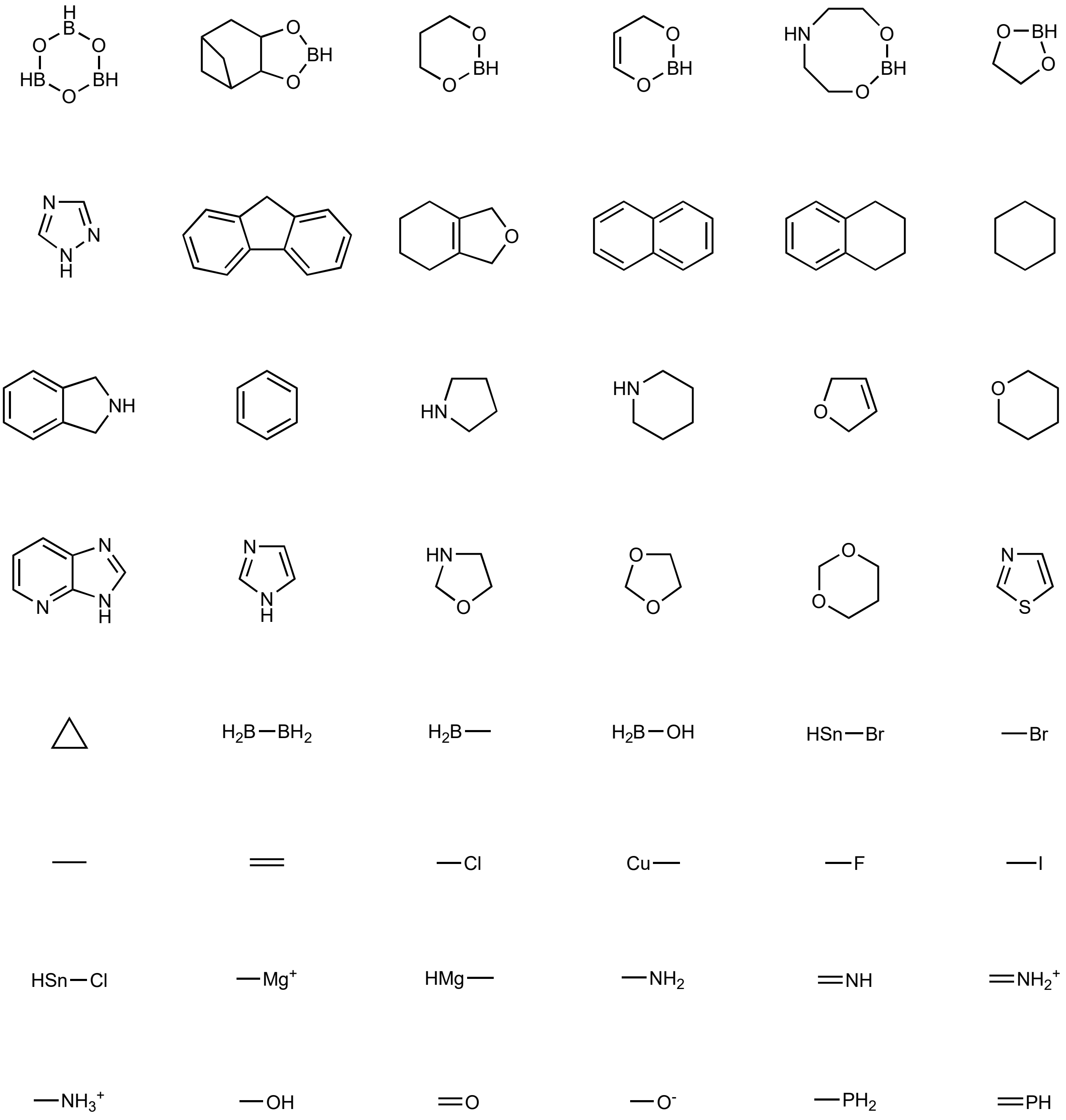

Supplementary Figure 3. 83 substructures that $\mathsf{G^2Retro}$ uses to complete synthons

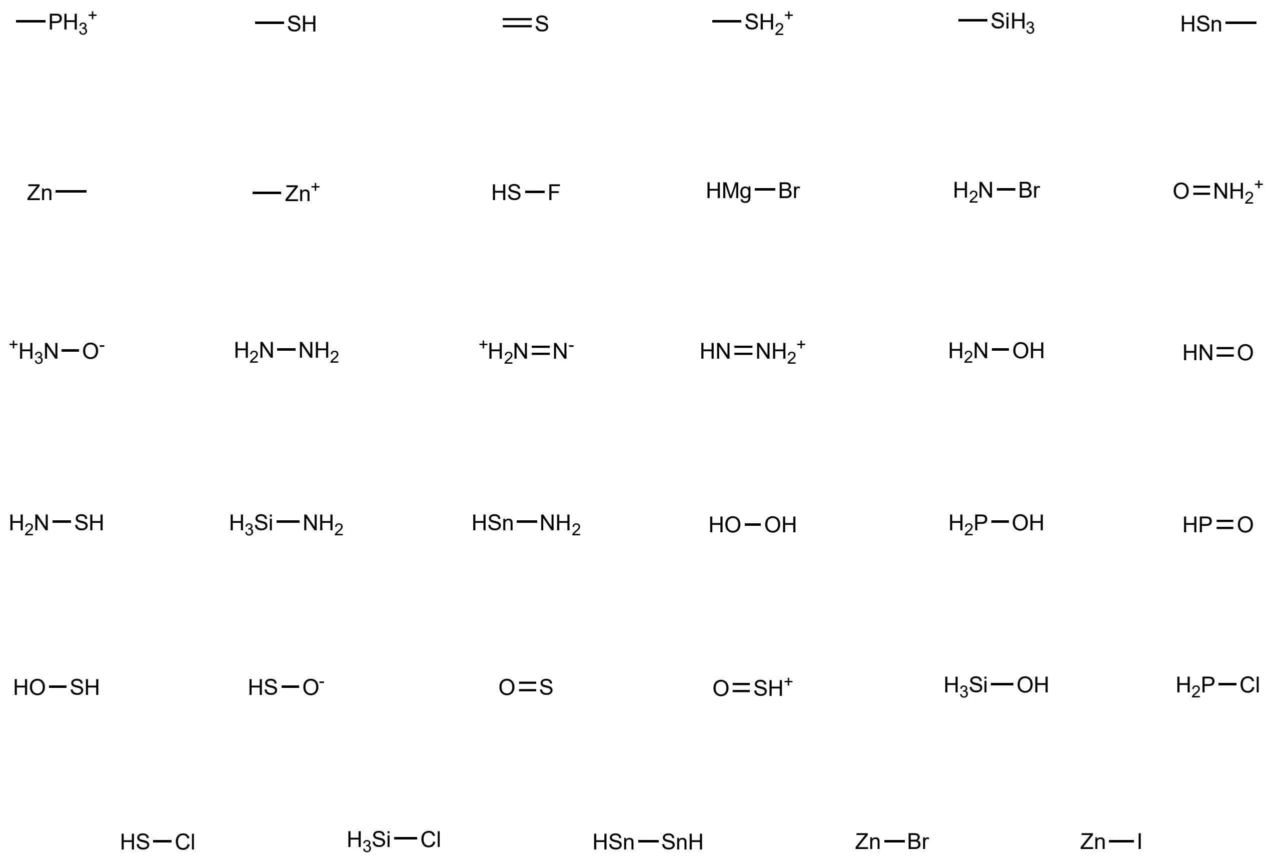

Supplementary Figure 4. 83 substructures that G^{2}Retro uses to complete synthons

Supplementary Note 8: Parameters for Reproducibility

We tuned the hyper-parameters of the reaction center identification module and the synthon completion module for G^{2}Retro and G^{2}Retro-B with the grid-search algorithm. We presented the parameter space in Supplementary Table 3. We determined the optimal hyper-parameters of the two modules for G^{2}Retro and G^{2}Retro-B according to the corresponding top-1 accuracy over the validation molecules.

Supplementary Table 3. Hyper-parameter space for G^{2}Retro and G^{2}Retro-B

Hyper-parameters	Values
hidden layer dimension	{128, 256, 512}
atom embedding dimension	{32}
# iterations of GMPN	{5, 7, 10}
# iterations of FMPN in G^{2}Retro-B	{3, 5, 7}

In the reaction center identification module, when reaction types are known, the optimal hidden dimension for G^{2}Retro and G^{2}Retro-B is 512; the optimal iterations of GMPN for G^{2}Retro and G^{2}Retro-B are 7 and 10, respectively; the optimal iteration of FMPN for G^{2}Retro-B is 7. When reaction types are unknown, the optimal hidden dimension for G^{2}Retro and G^{2}Retro-B is 512 and 256, respectively; the optimal iterations of GMPN for G^{2}Retro and G^{2}Retro-B are 5 and 10, respectively; the optimal iteration of FMPN for G^{2}Retro-B is 7. G^{2}Retro and G^{2}Retro-B share the same synthon completion model. In the synthon completion module, when reaction types are known, the optimal hidden dimension is 512; the optimal iteration of GMPN for G^{2}Retro is 5. When reaction types are unknown, the optimal hidden dimension is 512; the optimal iterations of GMPN is 7.

We optimized the models with batch size 256, learning rate 0.001 and learning rate decay 0.9. For the reaction center module, we trained the models for 150 epochs and checked the validation accuracy at the end of each epoch. We reduced the learning rate by 0.9 if the validation accuracy does not increase by 0.01 for 10 epochs. We saved the model with the optimal top-3 accuracy on reaction center identification over the validation dataset. For the synthon completion module, we trained the models for 100 epochs and checked the validation accuracy at the end of each epoch over 2,000 reactions that are randomly sampled from the validation set. We reduced the learning rate by 0.9 if the validation accuracy does not increase by 0.01 for 5 epochs. We saved the model with the optimal top-1 accuracy on synthon completion over the sampled subset of the validation dataset.

We implemented our models using Python-3.6.9, Pytorch-1.3.1, RDKit-2019.03.4 and NetworkX-2.3. We trained our models on a Tesla P100 GPU and a CPU with 32 GB memory on Red Hat Enterprise 7.7. The training of our reaction center identification model took $16 \sim 18$ hours, while the training of our synthon completion model took $32 \sim 34$ hours.

Supplementary References

1. Zhang, Z., Liu, Q., Wang, H., Lu, C. & Lee, C.-K. Motif-based graph self-supervised learning for molecular property prediction. In Ranzato, M., Beygelzimer, A., Dauphin, Y., Liang, P. & Vaughan, J. W. (eds.) *Advances in Neural Information Processing Systems*, vol. 34, 15870–15882 (Curran Associates, Inc., 2021).
2. Degen, J., Wegscheid-Gerlach, C., Zaliani, A. & Rarey, M. On the art of compiling and using 'drug-like' chemical fragment spaces. *ChemMedChem* **3**, 1503–1507 (2008).
3. Keam, S. J. Mavacamten: First approval. *Drugs* **82**, 1127–1135 (2022).
4. Oslob, J. *et al.* Pyrimidinedione compounds. US20170281626A1 (2017).
5. Hoy, S. M. Oteseconazole: First approval. *Drugs* **82**, 1017–1023 (2022).
6. Wirth, D. D., Yates, C. M. & Hoekstra, W. J. Antifungal compound process. WO2017049096A1 (2017).
7. Miyaura, N. & Suzuki, A. Palladium-catalyzed cross-coupling reactions of organoboron compounds. *Chem. Rev.* **95**, 2457–2483 (1995).
8. Fanta, P. E. The ullmann synthesis of biaryls. *Synthesis* **1974**, 9–21 (1974).
